# GaitSG: Gait Recognition with SMPLs in Graph Structure

**DOI:** 10.3390/s23208627

**Published:** 2023-10-22

**Authors:** Jiayi Yan, Shaohui Wang, Jing Lin, Peihao Li, Ruxin Zhang, Haoqian Wang

**Affiliations:** Tsinghua Shenzhen International Graduate School, Tsinghua University, Shenzhen 518055, China; yanjy21@mails.tsinghua.edu.cn (J.Y.); wang-sh21@mails.tsinghua.edu.cn (S.W.); lin-j21@mails.tsinghua.edu.cn (J.L.); lph21@mails.tsinghua.edu.cn (P.L.); zrx18@mails.tsinghua.edu.cn (R.Z.)

**Keywords:** gait recognition, 3D SMPL modality, graph neural network, robustness

## Abstract

Gait recognition aims to identify a person based on his unique walking pattern. Compared with silhouettes and skeletons, skinned multi-person linear (SMPL) models can simultaneously provide human pose and shape information and are robust to viewpoint and clothing variances. However, previous approaches have only considered SMPL parameters as a whole and are yet to explore their potential for gait recognition thoroughly. To address this problem, we concentrate on SMPL representations and propose a novel SMPL-based method named GaitSG for gait recognition, which takes SMPL parameters in the graph structure as input. Specifically, we represent the SMPL model as graph nodes and employ graph convolution techniques to effectively model the human model topology and generate discriminative gait features. Further, we utilize prior knowledge of the human body and elaborately design a novel part graph pooling block, PGPB, to encode viewpoint information explicitly. The PGPB also alleviates the physical distance-unaware limitation of the graph structure. Comprehensive experiments on public gait recognition datasets, Gait3D and CASIA-B, demonstrate that GaitSG can achieve better performance and faster convergence than existing model-based approaches. Specifically, compared with the baseline SMPLGait (3D only), our model achieves approximately twice the Rank-1 accuracy and requires three times fewer training iterations on Gait3D.

## 1. Introduction

Gait describes the walking pattern of a person. Unlike other biometrics, e.g., face, fingerprint, or iris, gait can be observed at a distance without the cooperation of the target. Thus, it is a perfect choice for criminal investigation and social security management. Gait recognition aims to signify the target person by learning his unique walking pattern through video sequences or pictures. It can be categorized into two types by input: appearance-based methods and model-based methods. The advantages and disadvantages of different gait modalities are shown in Table 1. The main-stream appearance-based methods [1,2,3,4,5] take silhouettes as input and achieve impressive performance on the in-the-lab scenarios [6,7]. However, being sensitive to clothing and viewpoint variances, these methods are more likely to lose their advantages in the uncontrolled environments. Model-based methods [8,9,10,11,12] use articulated human body representations (e.g., skeleton and SMPL) as inputs. These methods are robust to the carrying status and clothing variance because they focus on human body structure and movements. Among them, skeletons are sparse representations and are unaware of the human shape information, which is, unfortunately, a critical characteristic for identification. Therefore, a viewpoint-robust and dense representation is needed for accurate gait recognition.

The presence of the skinned multi-person linear (SMPL) model [13] makes it possible to break the above limitations. The SMPL model parameterizes the human mesh by 3D joint angles into low-dimension linear shape space and can implicitly provide dense 3D human mesh information. Hence, it is invariant to viewpoint and clothing interference, making it a more suitable representation of gait recognition. Zheng et al. introduce SMPLGait [11], which utilizes a multi-layer perceptron (MLP) network to extract SMPL features and then aggregates the SMPL features with silhouette features for gait recognition. They indicate that incorporating the SMPL modality can improve the accuracy of gait recognition. However, the effectiveness of using SMPL alone, without the additional input of silhouettes, has not been demonstrated yet. The dense shape information provided by silhouettes may impede the network’s ability to excavate the shape information from SMPLs. Moreover, SMPLGait considers SMPL parameters as a whole and learns SMPL features by using a simple MLP network, which may limit the expressiveness of SMPLs. Therefore, maximizing the potential of SMPLs remains open to ongoing research.

In response to the above problems, we focus on the representation learning of SMPL models and propose a novel framework for Gait recognition with SMPL representations in Graph structures, dubbed GaitSG. To concentrate on the behavior of SMPLs, we use the SMPL model as the sole input modality. We model them as graphs and introduce two graph-related vital components, the spatial feature extraction block (SFEB) and the part graph pooling block (PGPB), to unlock the potential of SMPLs. The SFEB is designed to learn spatial features from an SMPL model, where pose and shape features are learned, respectively. Inspired by the works of Teepe et al. [9,10], we consider the pose parameters of an SMPL model as graph nodes and propose the SFEB to extract discriminative features from SMPLs. Moreover, we introduce human body priors in PGPB through a novel part-splitting scheme, which enhances the viewpoint perception and maps the features into a more discriminative space. The main contributions of our work can be summarized as:

- We propose the first method that investigates the representation learning of SMPLs on gait recognition and demonstrates that expressive performance can be obtained with sole SMPL modality.
- We design a simple yet effective GCN-based block called SFEB to learn the human topology and extract SMPL features. SFEB not only improves the recognition accuracy but also leads to a quicker convergence.
- We propose a domain-inspired block, PGPB, to learn more discriminative gait features. PGPB introduces the human body prior by a novel part-splitting mechanism and can improve the accuracy and robustness.
- Thorough ablation studies prove the effectiveness of the proposed SFEB and PGPB. Evaluation results on influential benchmarks, the Gait3D dataset and the CASIA-B dataset, show the superiority of GaitSG over SOTA model-based methods.

The rest of the paper is organized as follows: a brief literature analysis of existing works is presented in Section 2; the proposed gait recognition method GaitSG is presented in Section 3; the performance evaluation of the proposed GaitSG is discussed in Section 4; and, finally, the discussion is summarized in Section 5.

## 2. Related Work

### 2.1. Gait Recognition

Gait recognition can be categorized into two main categories: appearance-based methods and model-based methods. The former typically employs silhouette sequences as inputs, while the latter employs human body models including skeletons and meshes.

#### 2.1.1. Silhouette-Based Gait Recognition

Silhouette-based methods rely on silhouettes obtained through background subtraction from videos. Early approaches [14,15,16,17] use gait energy images (GEIs) as a compressed representation of gait silhouette sequences. Recently, deep CNNs have been applied to learn gait representations [2,4,5,18], and demonstrated promising performance. For instance, GaitSet [2] proposes a set-pooling technique that regards a sequence of silhouettes as a set, thereby reducing the impact of unnecessary sequence order information. Lin et al. propose GaitGL [5] to exploit global and local features from frames. GLN [4] merges silhouette-level and set-level features in a top-down manner. Yuki H et al. [19] leverage an encoder-decoder structure to deform gait silhouette images from videos. Sheth A et al. [20] leverage a convolutional neural network consisting of eight layers to identify human gait. Dou H et al. [21] design a framework based on counterfactual intervention learning to focus on the regions that reflect effective walking patterns. Ma K et al. [22] propose DANet to simultaneously capture the global gait motion patterns and the local ones. Although silhouettes can provide informative appearance features, they may lose some information regarding the motion patterns and body structures of humans. Consequently, this modality is susceptible to clothing and viewpoint variances, especially for cases in the wild. Additionally, silhouettes are 2D representations and can be sensitive to viewpoints.

#### 2.1.2. Skeleton-Based Gait Recognition

Skeletons guarantee robustness against variations in clothing and viewpoint in gait recognition. Recent advances in human pose estimation have reached high accuracy, which have made skeleton-based approaches increasingly popular [8,9,10]. Liao et al. propose the pose-based temporal-spatial net (PTSN) [23], which leverages pose keypoints for gait recognition. PTSN incorporates a CNN to extract spatial features and an LSTM to extract temporal features. They further generate handcraft features from the skeleton keypoints, including joint angles, bone lengths, and joint motion, and then learn high-level features using a CNN [8]. Teepe et al. [9] model the human skeleton as a graph and use graph convolutional networks for gait recognition. They also combine higher-order inputs with residual networks [10]. Liu et al. [24] design a symmetry-driven hyper feature graph convolutional network to automatically learn multiple dynamic patterns and hierarchical semantic features. PoseMapGait [25] exploits the pose estimation maps to preserve rich clues of the human body and enhance robustness. Jun et al. [26] leverage a composition of the graph convolutional network, the recurrent neural network, and the artificial neural network to encode skeleton sequences, joint angle sequences, and gait parameters. Han et al. [27] propose a discontinuous frame screening module for the front end of the feature extraction part, to filter rich information. However, it is challenging to capture global appearance descriptions of a human using skeleton-based approaches.

#### 2.1.3. SMPL-Based Gait Recognition

The SMPL model can be a compelling modality as it overcomes the limitations of the two modalities discussed above. On the one hand, SMPLs record the keypoints of skeletons, which allows for a focus on the motion pattern of human gait. On the other hand, SMPLs include human shape information, which is crucial in distinguishing between individuals. Furthermore, the human shape information in SMPLs is of low dimension, making it less sensitive to human appearance variations. Li et al. [28] propose an end-to-end method for gait recognition through human mesh recovery (HMR), which is the first SMPL-based gait recognition method, and further exploit multi-view constraints to extract more consistent pose sequences [12]. However, they do not focus on human gait priors and lack illustrations of real-world performances. Zheng et al. introduce SMPLGait [11], which is based on accurate SMPL estimations. They use an MLP network to extract SMPL features and then aggregate them with silhouette features for gait recognition. However, these methods did not fully utilize the articulated characteristics of SMPLs and failed to capture the detailed relationships among joints.

To fulfill the above research gaps, we propose an SMPL-based framework for gait recognition. Specifically, we leverage the graph convolutional network and gait priors to unlock the potential of SMPLs.

### 2.2. 3D Human Reconstruction

The 3D human body can be represented in various ways, such as template parameters, meshes, voxels, UV position maps, and probabilistic outputs [29]. Currently, template parameters are the most widely used representation in the research community. A typical type of template parameters is the SMPL model [13], which is a vertex-based parametric model. The SMPL factors are deformed into shape and pose parameters. The shape parameters are obtained by performing the principal component analysis (PCA) in a low-dimensional shape space, which helps to prevent the gait recognition network from getting bogged down in silhouette details. The SMPL model depicts minimally clothed humans, allowing for the restoration of human body pose and appearance to a great extent and making it a favorable modality for gait recognition. Additionally, the SMPL family includes other models such as SMPL-X [30] and SMPL-H [31]. These models extend the SMPL model by including detailed hand poses and facial expressions. Notably, these features are redundant for gait recognition, and thus we use the SMPL model in this work and leave the extended models for future research.

## 3. Proposed Method

### 3.1. Pipeline Overview

**Figure 1:**
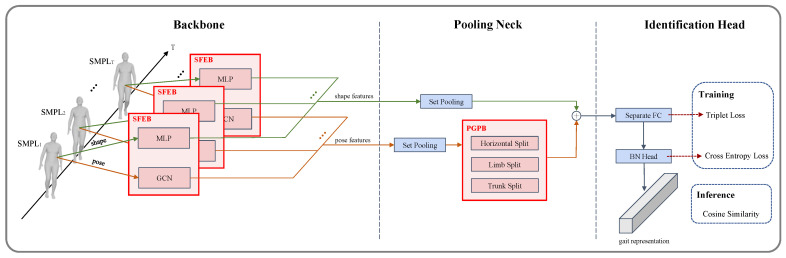
The overall architecture of the proposed GaitSG. Given a sequence of SMPL models of a person, spatial features of both shape and pose parameters are extracted, respectively, by an SFEB in each frame at the backbone stage. A pooling neck then aggregates the temporal features by regarding a video clip as a set. The features of the two branches are compressed, respectively, by a set pooling block. Specifically, as for the pose branch, PGPB concentrates on multi-scale features with three split techniques, including horizontal split, limb split, and trunk split. After that, an identification head with a seperate FC and a BN head maps the features into a discriminative space to obtain the gait representation.

**Figure 2:**
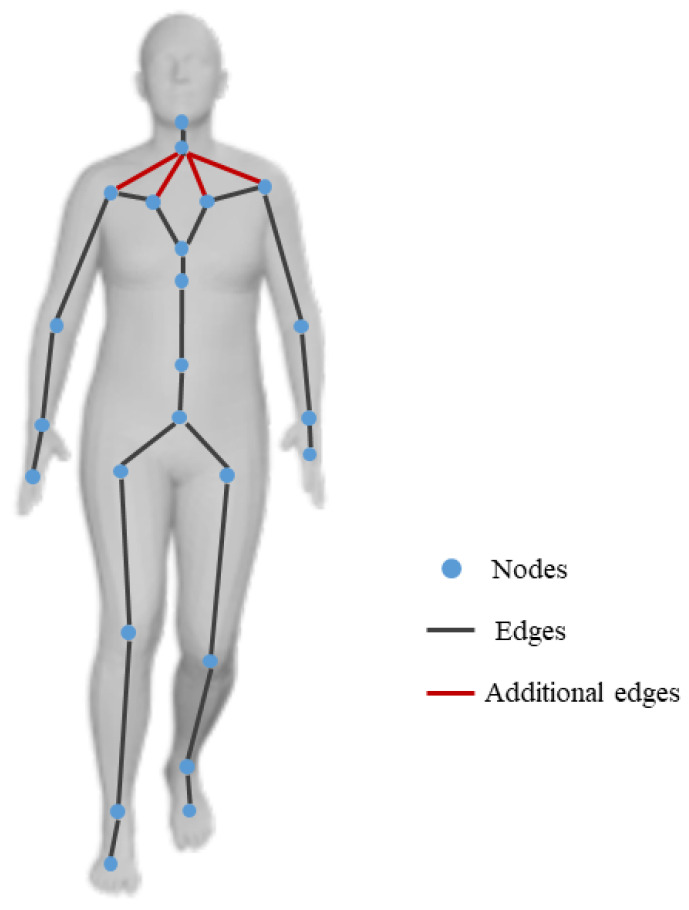
The graph structure of pose parameters.

The overall pipeline of GaitSG is shown in Figure 1 and the two designed blocks are shown in Figure 2. GaitSG takes SMPLs as inputs and regards them as graphs. Firstly, given a sequence of SMPL parameters, we use an SMPL feature extraction block (SFEB) to obtain high-dimensional shape and pose features spatially. In SFEB, shape features are extracted by an MLP network, while the pose parameter is regarded as a graph structure whose features are extracted by a GCN. Then, we aggregate the temporal features at the pooling neck with a set pooling block [2] and an elaborately designed part graph pooling block (PGPB). PGPB explores the gait representation by detecting relationships among the joints and the characteristics of SMPL models. Finally, the aggregated pose and shape features are combined by the element-wise addition operation and linearly projected to a metric space by an identification head for final gait identification.

### 3.2. SMPL Feature Extractor Block (SFEB)

The SMPL model of each frame consists of shape parameters $\beta \in R^{10}$ and pose parameters $\theta \in R^{3\times 24}$ [13]. Since pose parameters encode local information between adjacent joints, we use a graph structure, $G=\left(V,E\right)$, to represent θ in an SMPL model. $V=\left\{v_{1},\cdots ,v_{n}\right\}$ is the set of nodes representing joints, where n= 24. E is the set of edges, and $e_{ij}=\left(v_{i},v_{j}\right)\in E$ denotes the connection between $v_{i}$ and $v_{j}$. E is captured by an adjacency matrix $A\in R^{n\times n}$, where $A_{i,j}=1$ if $e_{ij}\in E$; otherwise, $A_{i,j}=0$. Given that there could be a motion pattern between these parts when people walk, we add four more edges to the graph between the neck node and the collarbone nodes to better simulate real-life scenarios, as is shown in Figure 2.

An SFEB is used to extract the spatial features of an SMPL model in each frame. In the SFEB, we design the networks according to the distinct data structures of the two groups of SMPL parameters. Shape features are learned by an MLP network (see SFEB in Figure 1 for detailed configuration). The MLP network consists of three linear layers, each of which is followed by batch normalization. Additionally, dropout is applied to the first two layers to avoid overfitting.

Motivated by Teepe et al. [9] and Kanazawa et al. [32], we use a GCN to learn pose features. The input tensor of the *ℓ*-th layer is denoted as $X^{\left(\ell \right)}$. For each frame in a video clip, the input θ is represented as a tensor with a dimension of (b,n×c), where *b* is the batch size, *n* is the number of joints in an SMPL model, and c=3 is the dimension of the rotation angle. We then apply batch normalization and modify it to the shape of (b,n,c). The modified tensor $X^{\left(0\right)}$ and the adjacency matrix A are then fed into the network. The GCN [33] is composed of three residual blocks, and each block can be calculated from layer *ℓ* to layer ℓ+1 as

(1) $$\begin{array}{c} X^{\left(\ell +1\right)}=\mathrm{ReLU}\left[X^{\left(\ell \right)}+\mathrm{GConv}\left(X^{\left(\ell \right)}\right)\right], \end{array}$$

where GConv(·)[34] represents the graph convolution. The update rule of GConv(·) is defined as

(2) $$\begin{array}{c} \mathrm{GConv}\left(X^{\left(\ell \right)},A\right)=S^{-\frac{1}{2}}AS^{-\frac{1}{2}}X^{\left(\ell \right)}W^{\left(\ell \right)}, \end{array}$$

where S is the diagonal degree matrix of A, and $W^{\left(\ell \right)}$ is the learnable weighted matrix at layer *ℓ*.

### 3.3. Part Graph Pooling Block (PGPB)

**Figure 3:**
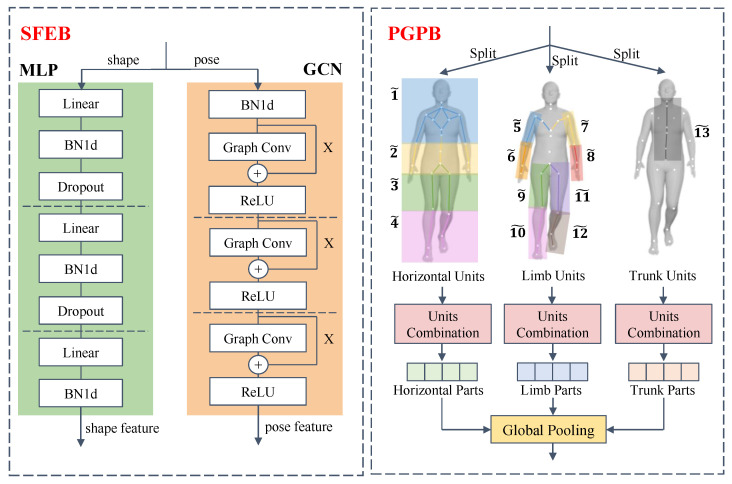
The detailed structure of SFEB. SFEB separates the input SMPL model into shape and pose parameters, and uses an MLP network and a GCN to extract their features, respectively. The detailed structure of PGPB. PGPB first generates units at horizontal level, limb level, and trunk level. Then, the units are combined into multi-scale parts. Each part produces a feature through the global pooling function.

Motivated by Thakkar et al. [35], in the PGPB, we design three splits to learn the different meanings of body parts. The splits are designed to resolve the physical distance-unaware limitation of the body graph structure. The distance between two nodes in a graph is defined as the minimum number of edges connecting them. Therefore, the physical Euclidean distance between human body joints is not completely preserved in the graph structure, i.e., the nodes can be near inside a human body but far in the pose graph, e.g., the nodes on the two legs. Additionally, since the graph convolution technique has a local receptive field, long-range dependencies in the graph can hardly be captured. To address the limitations, we propose a novel part-splitting mechanism to explicitly take the spatial position of the joints into consideration, and capture long-range dependencies on a human body, as shown in PGPB in Figure 3.

In each split, we first partition the nodes into several basic units. Each unit is composed of several predefined nodes, and $\tilde{i}$ denotes the *i*-th unit. In the first split, we divide the nodes in a body graph horizontally into four units: $\tilde{1}$, $\tilde{2}$, $\tilde{3}$, and $\tilde{4}$. Then, inspired by the multi-scale representation in the field of person re-identification [36], we apply a horizontal combination technique to the units, and group the units into parts in a multi-scale manner as shown in Table 2. We use a tuple $p_{k}=\left(\tilde{m},\tilde{n},...\right)$ to represent the *k*-th part. We set the scale to 3 to integrate the units. The units are contiguously integrated into different numbers of parts according to the scale $s\in \left\{1,2,4\right\}$. For example, if s=2, then the units are formed into two parts: $\left(\tilde{1},\tilde{2}\right)$ and $\left(\tilde{3},\tilde{4}\right)$. When s=4, there is only one part on this scale that represents the spatial graph as a whole. In the second split, we focus on limb patterns. Outer limbs are found to be important by Teepe et al. [10]. Based on their findings, we expand the scope to the whole limbs of the human body and study the features in detail with our unit-part technique. We identify eight limb units (see PGPB in Figure 3) and divide the units into 20 parts with three scales. The parts are defined in Table 2. In the third split, we concentrate on the trunk and identify it as a unit. The orientation of the human trunk mainly determines the walking direction of a person. The node at the pelvis, as the root in the human kinematic tree, denotes the global translation and rotation of a human body to the camera. Please note that the global orientation can be a substitute for the pelvis node. For the purpose of improving the robustness of human movements, we also take all the other nodes in a trunk besides the pelvis. We naturally assume that pose parameters on these nodes encode the viewpoint of the person. By this split, we can reduce the affection of camera views.

Finally, each part in the above splits generates a feature by the global pooling function [2] denoted as GP(·). The process of GP(·) applied on part $p_{k}$ is formulated as $\mathrm{GP}\left(p_{k}\right)=\mathrm{maxpool}\left(p_{k}\right)+\mathrm{avepool}\left(p_{k}\right)$, where maxpool(·) and avepool(·) denote global max pooling and global average pooling, respectively.

### 3.4. Identification Head

For ease of verifying the superiority of SFEB and PGPB, we adopt the same identification head as SMPLGait [11]. The head has a fully connected (FC) layer computed at the part level, denoted as seperate FC, and a batch-norm (BN) neck to generate gait representation features. The loss function has two components and is defined as

(3) $$\begin{array}{c} L=\alpha L_{tri}+\beta L_{ce}, \end{array}$$

where $L_{tri}$ is the triplet loss and $L_{ce}$ is the cross-entropy loss. α and β are the hyper-parameters. The cosine similarity is adopted to measure the similarity between each pair of query and gallery sequences for testing.

## 4. Experiments

### 4.1. Datasets and Evaluation Metrics

**Gait3D** [11] is a recent challenging large-scale dataset. To the best of our knowledge, it is the only outdoor dataset that provides accurate 3D SMPL gait representations. Moreover, it contains 4k subjects and over 25k sequences from 39 cameras in the wild. There are conditions in the real world, including viewpoint variance and occlusion cases, and people walking at varying speeds and routes.

**CASIA-B** [6] is a popular in-the-lab dataset that provides RGB videos. It contains 124 subjects and captures sequences through 11 views and three walking conditions. The conditions include normal (NM), walking with a bag (BG), and wearing a coat or jacket (CL). We use the same human reconstruction method as the Gait3D dataset, ROMP [37], to generate SMPL models by frame, and then discard low-quality predictions.

**Evaluation metrics:** For Gait3D, we report the average Rank-1 (R-1) and Rank-5 (R-5) accuracy rates, the mean average precision (mAP), and the mean inverse negative penalty (mINP) over all query sequences. MAP and mINP consider the retrieval performance, in which the latter focuses more on hard samples. For CASIA-B, we report the R-1 score for all views of different types and the average score across views.

### 4.2. Implementation Details

We set the dropout ratio in the SFEB as 0.2 for GaitSG and 0.3 for GaitSG+. The hyper-parameters in Equation (3) are set as α=1 and β=0.1, the same as SMPLGait [11]. Our models are trained by the ADAM optimizer with a weight decay of $5\times 10^{-4}$. In Gait3D, we train GaitSG with 50k iterations and GaitSG+ with 40k iterations. The learning rate is initialized as $1\times 10^{-4}$ and then multiplied by 0.1 at the iteration of 32k. In CASIA-B, the iterations are 10k, and the learning rate is $1\times 10^{-3}$ for the two models. Our codebase is OpenGait [38]. The experiments are conducted on two NVIDIA GeForce GTX 2080 Ti GPUs.

### 4.3. SOTA Methods

We compare our model with several model-based gait recognition algorithms, including four skeleton-based methods and an SMPL-based method.

**PoseGait**: PoseGait exploits human 3D pose for gait recognition which is defined by the 3D coordinates of joints of the human body.

**GaitGraph**: GaitGraph combines skeleton poses with a graph convolutional network to obtain a modern model-based approach for gait recognition.

**PoseMapGait**: PoseMapGait exploits the pose estimation maps, which are decomposed as one heatmaps evolution feature and one pose evolution feature.

**SDHF-GCN**: SDHF-GCN addresses the skeleton-based gait recognition task with a symmetry-driven hyper feature graph convolutional network. The model involves three dynamic patterns: natural connection, temporal correlation, and symmetric interaction, which enriches the description of dynamic patterns by exploiting symmetry perceptual principles.

**SMPLGait**: SMPLGait explores dense 3D representations for gait recognition in the wild. It simultaneously uses both silhouettes and the SMPL models as inputs.

### 4.4. Comparisons with Other Methods

We modify SMPLGait to keep the 3D-STN branch only for fair comparisons and take it as our baseline. We also establish GaitSG+, which replaces the GCNs in GaitSG with ST-GCNs (spatial temporal graph convolutional networks) [39,40] to extract temporal features in adjacent frames of the same joint at the backbone stage.

Table 3 demonstrates that GaitSG and GaitSG+ outperform other SOTA methods in three out of the four metrics on Gait3D. Specifically, our original model (GaitSG) surpasses the best-performance method (GaitGraph) by 0.15% R-1, 0.91% mAP, and 1.87% mINP, and GaitSG+ by 1.15% R-1, 0.35% mAP, and 1.11% mINP. The comparison between GaitSG and GaitSG+ indicates that temporal information is rich at the backbone stage, and temporal features are hard to learn in unconstrained real-world scenarios. Moreover, the GCN-based methods, including GaitGraph and ours, obtain higher accuracy, showing that the GCN technique can effectively capture gait features and enhance the overall accuracy performance. Additionally, our GaitSG and GaitSG+ exceed the baseline method, SMPLGait (3D only), by a large margin, which verifies the improvement on the accuracy of our SFEB and PGPB. Meanwhile, our SMPL-based methods marginally outperform skeleton-based methods. The results indicate that the introduction of shape information has a positive impact on gait recognition tasks. However, the benefit of shape information is limited because shape parameters are of low dimension, and the accuracy of SMPL generation methods remains to be further improved.

**Figure 4:**
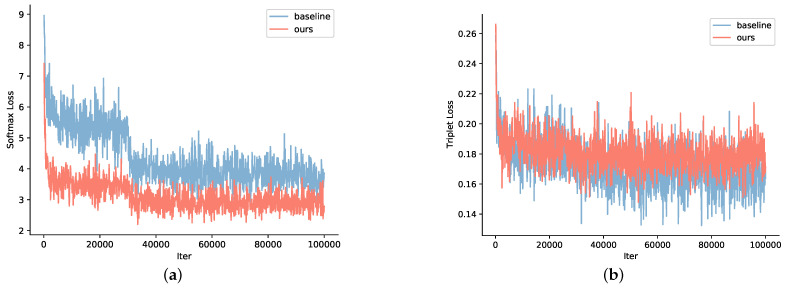
Comparison of training loss curves between our GaitSG and the baseline on Gait3D. (**a**) Softmax loss. (**b**) Triplet loss.

We also perform experiments on convergence comparison between GaitSG and the baseline in Gait3D. Figure 4 indicates that GaitSG has faster convergence. As is shown in Figure 4a, the curve of our method can reach a lower softmax loss at an early stage and remains stable after 32k iterations, while the baseline curve has two sharp drops and slowly declines to convergence. The drops are caused by the decrease in the learning rate. The triplet loss curves for the two methods are shown in Figure 4b. The convergence trends and the loss values are similar, while our method has a relatively minor loss variance. Experimentally, our framework requires three times fewer iterations than the baseline method. The results indicate that considering the SMPL as a graph structure instead of a simple tensor structure allows the model to capture gait features more efficiently.

Table 4 illustrates the improvement in accuracy of our framework on the CASIA-B dataset, which is effective in all of the three types and most of the 11 views. From the condition perspective, GaitSG reaches higher scores in normal conditions (NM), with 91.7% mean R-1 and bag-carrying conditions (BG) with 77.1% mean R-1, while GaitSG+ performs better in cross-clothing conditions (CL), with 68.4% mean R-1. Additionally, from the view perspective, GaitSG+ surpasses GaitSG in one view of NM condition, four views of BG condition, and nine views of CL condition. These findings indicate that temporal feature learning at the backbone stage can enhance the robustness of the framework in some severe but certain conditions.

### 4.5. Ablation Study

To verify the effectiveness of our proposed two key modules, the SMPL feature extractor block (SFEB) and the part graph pooling block (PGPB), we adopt the Gait3D dataset to conduct the following ablation studies:

#### 4.5.1. Different Inputs and Networks in the SFEB

Table 5 shows the influence of different inputs and backbones in the SFEB. As for the input, we find that feeding both pose and shape parameters into the model can achieve higher scores than using either one of them, which indicates the effectiveness of making full use of SMPL parameters rather than using only some of them. By analyzing the performances of the first three methods, we observe that pose parameters contribute more than shape parameters to the four scores. There are mainly two reasons. The first reason is that actions are more important than appearances in gait recognition. In our experiment, pose parameter sequences encode the walking way, which contributes more to the gait recognition task, while shape parameters encode the human appearance and thus have a weaker influence. The second reason is that the number of input features matters. In an SMPL model, pose parameters have 72 dimensions, while shape parameters have only 10 dimensions. The dimension of pose parameters is significantly higher than that of shape parameters; accordingly, pose parameters play a more important role.

As for the feature extraction module, the graph convolution technique has more potential than the MLP network in extracting pose parameter features. Moreover, we further apply ST-GCNs instead of GCNs. The R-1 and R-5 scores increase, while the mINP and mINP scores decrease. One possible reason is that the temporal graph convolution can destroy outlier patterns and remove some details, leading to the insignificance of the gait feature in hard samples.

#### 4.5.2. Different Splits in the PGPB

We conduct a break-down ablation to investigate the splits in the PGPB. The results are listed in Table 6. All of these experiments apply GCNs in the SFEB. We observe that the whole body feature equipped with the horizontal split can achieve 5.30% R-1, 12.70% R-5, 4.98% mAP, and 3.31% mINP. When we further apply the limb split, the model achieves 0.6%, 0.4%, 0.42%, and 0.28% improvements in R-1, R-5, mAP, and mINP, respectively. Then, we add the trunk split, and the model continuously gains by 0.5% R-1, 0.9% R-5, 0.65% mAP, and 0.7% mINP. These results indicate the effectiveness of the splits.

#### 4.5.3. Viewpoint Encoding

An SMPL model, as a 3D representation, has the advantage of alleviating the viewpoint variance in gait recognition. We explicitly capture the viewpoint information and analyze the influence of different encoding methods in Table 7. Method A uses our framework without encoding any view information. Method B exploits the previous scheme [11] that takes camera parameters as additional input. We need actual camera parameters to alleviate the view problem, that is, to shift an SMPL from one camera perspective to another. However, camera parameters provided in the Gait3D dataset are weak perspective projection matrices. These parameters can only transform 2D images into 3D coordinates, or reproject 3D representations into image coordinates, which fail to meet our need. For this reason, camera parameters provided by Zheng et al. are unnecessary information within our framework. We explain the limited improvement derived from the error reduction between the estimated and the actual camera parameters for comprehensiveness. Method C denotes our method, which adds the trunk split by assuming the trunk nodes encode the viewpoint. Our method surpasses method A by a large margin. It verifies that our method can play a role in alleviating the viewpoint variance issue.

## 5. Discussion

Compared with silhouette-based methods, our SMPL-based method, GaitSG, seems to have little advantage over accuracy. The main reason is that the upstream SMPL generation technique is not yet as mature as was expected, which could significantly affect the overall performance. However, we are confident that the emergence of a higher dimensional representation of SMPL model with more detailed descriptions of pose and shape, which is a promising subfield in the near future, could further release the potential of GaitSG on a broader range of tasks.

Another limitation is that gait datasets in SMPL modality are scarce. To the best of our effort, we could only find one public dataset, the Gait3D, which has accurate SMPLs. CASIA-B provides RGB videos that can generate SMPLs. Hence, we conduct experiments only on these two datasets. As SMPLs intuitively suit real-world scenarios more than other modalities, we sincerely hope that more datasets would provide the SMPL modality to promote this community. GaitSG has improved performance on accuracy and convergence while taking the sole SMPL modality as input. Future work will consider incorporating advanced graph techniques and fusing with other gait modalities for better identification.

In this paper, we propose a novel model-based gait recognition framework, GaitSG, which is the first attempt to study intensively on SMPL representations for gait recognition. To reduce the impact of other gait modalities, we use SMPL models as the only input type. We present the pose parameters as a graph and design the SFEB, which applies the graph convolution technique. In addition, we design the PGPB, which includes three splits to leverage human body priors and efficiently learns local and global features. The PGPB can also alleviate the limitation of the body graph and the viewpoint variance. Extensive experiments indicate the superiority in terms of accuracy and the quick convergence of GaitSG over other SOTA model-based methods. Specifically, our framework requires three times fewer training iterations than the baseline method SMPLGait (3D only). GaitSG achieves 6.4% R-1 accuracy, 14% R-5 accuracy, 6.09% mAP, and 4.29% mINP, while GaitSG+ achieves 7.4% R-1 accuracy, 14.6% R-5 accuracy, 5.53% mAP, and 3.53% mINP on Gait3D. The baseline method SMPLGait (3D only) achieves 4.30% R-1 accuracy, 13.20% R-5 accuracy, 5.10% mAP, and 3.30% mINP on Gait3D. GaitSG achieves 91.7%, 77.1%, and 66.8% mean accuracy in NM, BG, and CL conditions of CASIA-B, respectively, while the baseline method achieves 84.5%, 70.9%, and 64.2% on the three conditions. The performance verifies that the potential of SMPL modality can be fully reached within our framework.

## Figures and Tables

**Table 1:** Advantages and disadvantages of different gait modalities. The representations are taken from the same person at the same timestamp in the Gait3D dataset.

	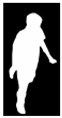	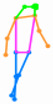	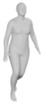
Type	Silhouette	Skeleton	SMPL
Cloth	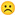 Sensitive	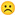 Sensitive	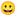 Robust
Viewpoint	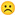 Sensitive	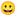 Robust	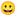 Robust
Shape	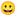 Easy	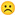 Hard	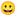 Easy
Space	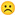 2D	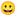 2D/3D	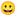 3D

**Table 2:** The parts of different splits in an SMPL model are presented in the list format. Scale is the number of units in a part, and Total represents the number of the parts in a split with all scales.

Scale	Split1	Split2	Split3
1	$\left\{\left(\tilde{1}\right),\left(\tilde{2}\right),\left(\tilde{3}\right),\left(\tilde{4}\right)\right\}$	$\left\{\left(\tilde{5}\right),\left(\tilde{6}\right),\left(\tilde{7}\right),\left(\tilde{8}\right),\left(\tilde{9}\right),\left(\tilde{10}\right),\left(\tilde{11}\right),\left(\tilde{12}\right)\right\}$	$\left\{(\tilde{13})\right\}$
2	$\left\{\left(\tilde{1},\tilde{2}\right),\left(\tilde{3},\tilde{4}\right)\right\}$	$\left\{\left(\tilde{5},\tilde{6}\right),\left(\tilde{7},\tilde{8}\right),\left(\tilde{9},\tilde{10}\right),\left(\tilde{11},\tilde{12}\right),\left(\tilde{10},\tilde{12}\right)\right\}$	∖
4	$\left\{(\tilde{1},\tilde{2},\tilde{3},\tilde{4})\right\}$	$\left\{\left(\tilde{5},\tilde{6},\tilde{7},\tilde{8}\right),\left(\tilde{9},\tilde{10},\tilde{11},\tilde{12}\right),\left(\tilde{5},\tilde{6},\tilde{9},\tilde{10}\right),\left(\tilde{7},\tilde{8},\tilde{11},\tilde{12}\right)\right\}$	∖
Total	7	20	21

**Table 3:** Comparison of different model-based gait recognition methods on Gait3D. The maximal scores are marked in **bold**.

Methods	Input Type	R-1 (%)	R-5 (%)	mAP (%)	mINP (%)
PoseGait [8]	skeleton	0.24	1.08	0.47	0.34
GaitGraph [9]	skeleton	6.25	**16.23**	5.18	2.42
SMPLGait (3D only) [11]	SMPL	4.30	13.20	5.10	3.30
GaitSG (ours)	SMPL	6.40	14.00	**6.09**	**4.29**
GaitSG+ (ours)	SMPL	**7.40**	14.60	5.53	3.53

**Table 4:** Comparison of different model-based methods on CASIA-B. The results include the R-1 accuracies of each probe view and the average accuracies excluding identical-view cases. The maximal scores are marked in **bold**.

Gallery NM # 1-4		0°–180°											Mean
Probe		0°	18°	36°	54°	72°	90°	108°	126°	144°	162°	180°	
NM # 5-6	PoseGait	55.3	69.6	73.9	75.0	68.0	68.2	71.1	72.9	76.1	70.4	55.4	68.7
	PoseMapGait	59.9	76.2	81.7	83.1	76.8	76.1	76.3	81.1	79.6	75.4	66.1	75.7
	SDHF-GCN	77.3	82.8	85.1	86.0	85.5	85.4	83.7	81.5	80.5	83.9	77.6	82.7
	GaitGraph	85.3	88.5	91.0	92.5	87.2	86.5	88.4	89.2	87.9	85.9	81.9	87.7
	SMPLGait (3D only)	69.7	82.3	90.1	90.1	85.2	84.6	86.4	89.5	87.9	85.8	78.0	84.5
	GaitSG (ours)	**90.9**	**93.5**	**95.0**	**93.0**	88.9	**91.2**	**92.7**	**92.2**	**93.4**	**93.1**	**85.0**	**91.7**
	GaitSG+ (ours)	86.3	89.3	90.3	89.6	**89.4**	88.7	89.6	86.9	86.6	87.1	79.1	87.5
BG # 1-2	PoseGait	35.3	47.2	52.4	46.9	45.5	43.9	46.1	48.1	49.4	43.6	31.1	44.5
	PoseMapGait	47.7	56.1	63.9	63.3	64.2	59.5	58.1	61.5	61.9	58.2	44.3	58.1
	SDHF-GCN	67.5	73.9	73.2	74.3	68.5	68.5	70.5	69.0	62.2	68.7	60.1	68.8
	GaitGraph	75.8	76.7	75.9	76.1	71.4	**73.9**	78.0	74.7	**75.4**	**75.4**	69.2	74.8
	SMPLGait (3D only)	63.4	73.9	79.7	73.2	69.8	65.1	70.8	71.3	71.5	70.7	70.8	70.9
	GaitSG (ours)	76.5	**81.2**	**82.2**	**81.6**	76.2	70.6	**83.3**	**79.3**	**76.3**	72.5	68.9	**77.1**
	GaitSG+ (ours)	**80.2**	79.5	77.2	76.9	**78.7**	**73.9**	78.5	73.5	73.3	71.9	**75.0**	76.2
CL # 1-2	PoseGait	24.3	29.7	41.3	38.8	38.2	38.5	41.6	44.9	42.2	33.4	22.5	36.0
	PoseMapGait	30.4	41.9	45.2	48.9	47.3	48.1	46.5	44.9	36.0	34.5	29.6	41.2
	SDHF-GCN	63.4	65.4	66.7	64.8	63.0	66.2	69.1	63.3	61.1	65.9	60.7	64.5
	GaitGraph	**69.6**	66.1	68.8	67.2	64.5	62.0	69.5	65.6	**65.7**	66.1	**64.3**	66.3
	SMPLGait (3D only)	44.4	62.4	67.7	72.8	69.7	64.1	67.4	66.0	61.6	68.2	61.5	64.2
	GaitSG (ours)	60.9	68.1	70.6	70.3	69.7	64.6	67.5	63.4	64.0	**74.3**	61.5	66.8
	GaitSG+ (ours)	68.6	**70.7**	**74.5**	**75.5**	**72.4**	**68.4**	**70.0**	**69.3**	64.9	64.6	53.9	**68.4**

**Table 5:** Performance comparison of different SMPL parameters and backbones. The maximal scores are marked in **bold**.

	Pose			Shape	R-1 (%)	R-5 (%)	mAP (%)	mINP (%)
	MLP	GCN	ST-GCN	MLP				
A	✔				3.90	11.60	4.45	2.83
B		✔			6.00	13.30	6.04	4.19
C			✔		6.30	13.80	5.50	3.51
D				✔	0.60	1.30	0.40	0.24
E	✔			✔	4.70	12.40	4.60	2.89
F (ours)		✔		✔	6.40	14.00	**6.09**	**4.29**
G (ours)			✔	✔	**7.40**	**14.60**	5.53	3.53

**Table 6:** Performance comparison of different splits. The maximal scores are marked in **bold**.

	Horizontal	Limb	Trunk	R-1 (%)	R-5 (%)	mAP (%)	mINP (%)
A	✔			5.30	12.70	4.98	3.31
B	✔	✔		5.90	13.10	5.40	3.59
C (ours)	✔	✔	✔	**6.40**	**14.00**	**6.09**	**4.29**

**Table 7:** Performance comparison of the influence of view-encoding methods. The maximal scores are marked in **bold**.

	R-1 (%)	R-5 (%)	mAP (%)	mINP (%)
A (w/o view encoding)	5.90	13.10	5.40	3.59
B (A + Cam para input)	6.10	13.30	5.44	3.52
C (A + Trunk split, ours)	**6.40**	**14.00**	**6.09**	**4.29**

## Data Availability

The datasets in the paper are publicly available.
